# GERONTOLOGISTS’ CONSIDERATIONS IN CHOOSING AND EXPERIENCING LATER-LIFE RELOCATION AND RESIDENCE

**DOI:** 10.1093/geroni/igz038.814

**Published:** 2019-11-08

**Authors:** Helen Q Kivnick, Anne Wyatt-Brown, Helen Q Kivnick

**Affiliations:** 1 University of Minnesota School of Social Work, Saint Paul, Minnesota, United States; 2 University of Florida, Gainesville, Gainesville Florida, United States

## Abstract

Increasing numbers of Baby Boomers and their older cohorts are now confronting challenges of aging that have long been research topics and practice domains of gerontologists. Choosing later-life relocation and residence is one such challenge. However, large-scale, population-based research runs the risk of making unwarranted assumptions about individuals based on group membership, alone. As people continue to develop through older adulthood, residential preferences and priorities can change over time – based on financial pressures, health concerns, family circumstances, and more. No solution works for all people. And for many, a choice made early in older adulthood may prove unworkable in the longer term. As Stephen Golant put it in Aging in the Right Place (2015), it is important to try to match the place -- be it home, community, or institution -- with the temperament and the finances of the person who will be living in it. Each older adult needs information about different options and appropriate self-knowledge, in order make a decision that gives them pleasure for an extended amount of time. In this session, aging gerontologists (aged 69-90+) discuss their personal considerations in anticipating and making these major decisions and in experiencing their consequences – as influenced by the unique disciplinary and theoretical perspectives afforded by a lifetime of work in gerontology. Presenters will share their personal later-life housing stories, as they are influenced by theories, concepts, findings, personal circumstances, and styles of observing, thinking, and acting – all developed through their personal careers in gerontology.

